# Affecting Structure Characteristics of Rotary Swaged Tungsten Heavy Alloy Via Variable Deformation Temperature

**DOI:** 10.3390/ma12244200

**Published:** 2019-12-13

**Authors:** Adéla Macháčková, Ludmila Krátká, Rudolf Petrmichl, Lenka Kunčická, Radim Kocich

**Affiliations:** Faculty of Materials Science and Technology, VŠB–Technical University of Ostrava, 17. listopadu 15, 708 00 Ostrava, Czech Republic; adela.machackova@vsb.cz (A.M.); rudolf.petrmichl@vsb.cz (R.P.);

**Keywords:** tungsten heavy alloy, rotary swaging, finite element analysis, deformation behaviour, residual stress

## Abstract

This study focuses on numerical prediction and experimental investigation of deformation behaviour of a tungsten heavy alloy prepared via powder metallurgy and subsequent cold (20 °C) and warm (900 °C) rotary swaging. Special emphasis was placed on the prediction of the effects of the applied induction heating. As shown by the results, the predicted material behaviour was in good correlation with the real experiment. The differences in the plastic flow during cold and warm swaging imparted differences in structural development and the occurrence of residual stress. Both the swaged pieces exhibited the presence of residual stress in the peripheries of W agglomerates. However, the NiCO matrix of the warm-swaged piece also exhibited the presence of residual stress, and it also featured regions with increased W content. Testing of mechanical properties revealed the ultimate tensile strength of the swaged pieces to be approximately twice as high as of the sintered piece (860 MPa compared to 1650 MPa and 1828 MPa after warm and cold swaging, respectively).

## 1. Introduction

For their exceptional mechanical (high strength) and physical (high density, melting point, etc.) properties, tungsten heavy alloys (THAs) are primarily used to shield radiation or to block kinetic energy [1,2]. Nevertheless, they can also be used advantageously for other demanding applications, such as for production of therapeutic devices in oncology, for kinetic penetrators in the military, or as aircraft counterbalances [3,4]. THAs are typically produced via powder metallurgy—they are isostatically pressed and subsequently sintered at temperatures between 1000 and 1500 °C—from powder mixtures containing a majority (over 90 wt.%) of tungsten, in addition to other alloying elements featuring lower melting temperatures (e.g., Ni, Co, Fe), the boundaries of the grains of which melt during sintering and ensure binding of the tungsten particles and elimination of porosity via diffusion [5].

To enhance the strength of THAs, the tungsten content can be increased to 98 wt.%; however, the increase in strength is typically at the expense of plasticity. Additions of alloying (i.e., binding) elements generally decrease the strength, but increase plasticity. Contents of the alloying elements (i.e., matrix) lower than 3 wt.% can cause brittleness of the final product [6]. On the other hand, high contents of alloying elements contribute to inhomogeneity of the mechanical properties and usually also to an uneven shape of the sintered piece cross-section due to gravity sedimentation during sintering. Under optimized conditions, the matrix is homogenously distributed in the gaps between the tungsten agglomerates [7].

In addition to modification/optimization of their chemical composition, the mechanical properties of THAs can also be enhanced by post-sintering deformation processing, which can advantageously be performed via methods of severe plastic deformation [8,9,10]. Technologies applying intensive plastic deformation have recently gained attention primarily due to their ability to effectively refine the structural units and consequently enhance the mechanical and utility properties of the processed materials. Severe plastic deformation (SPD) methods, such as equal channel angular pressing (ECAP) [11,12,13], twist channel (multi) angular pressing (TCAP, TCMAP) [14,15], or high pressure torsion (HPT) [16], have been proven to be very effective; however, they are discontinuous and are only applicable for limited volumes of material. On the other hand, methods such as ECAP-conform [17,18], accumulative roll bonding (ARB) [19,20], or rotary swaging (RS) [21,22] are continuous and can favourably be used to manufacture relatively large (semi)products. Given its versatility, the RS technology is applicable in various industrial branches.

Rotary swaging is an incremental process, i.e., it is characterized by gradual increments of the imposed strain provided by high-frequency strokes of the swaging dies. The method can be applied under cold, as well as warm and hot conditions and is used to process solid work pieces, but also to produce hollow tubes and shaped axisymmetric products [23,24,25]. The processed materials are typically conventionally cast alloys, such as magnesium [26] and aluminium alloys [27]. Nevertheless, the favourable stress state and incremental character enables the method to advantageously be applied also to process composites, the example of which can be mechanically bonded Cu/Al clad composites for electrotechnic applications [28,29], powder-based materials, demanding compounds based on zirconium [30], or tungsten heavy alloys [31].

The aim of this work was to investigate material behaviour of the studied WNiCo tungsten heavy alloy during cold and warm rotary swaging, and the effects of the individual processing steps on its structure and properties. To better characterize the influence of the used technology, especially the effect of the applied pre-swaging induction heating, experimental investigations were supplemented with finite element analyses. The predicted results were verified by real swaging experiments. Attention was also given to characterization of residual stress within the structure.

## 2. Materials and Methods

### 2.1. Numerical Prediction

**Figure 1:**
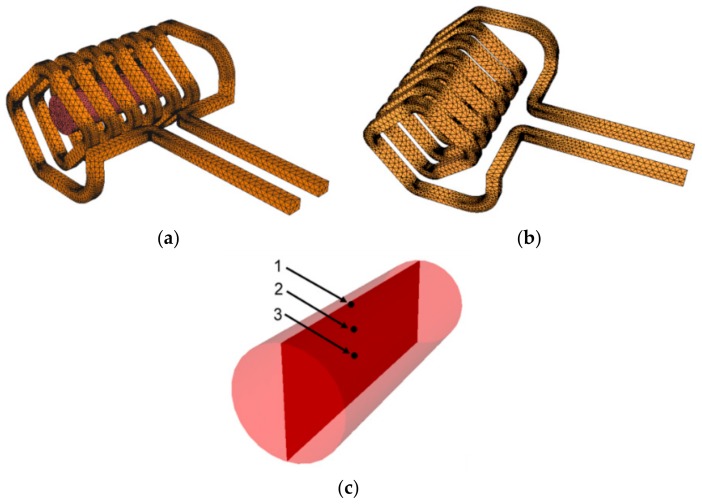
Schematic depiction of setup of induction heating, meshed (**a**); inductor with mesh (**b**); axial longitudinal cut through work piece with depicted monitored sensors 1, 2, and 3 (**c**).

The first step of the performed study was the numerical prediction of the applied induction heating process, which was performed via Finite Element Method (FEM) using FORGE (NxT version, Transvalor, Sophia Antipolis, France) software. The used computational model was a model based on the Maxwell’s equations available in the software. The prediction was implemented primarily to optimize control of the heating process and determine the heating time necessary to achieve homogeneous temperature distribution throughout the work piece cross-section before the warm rotary swaging, i.e., to predict behaviour of the studied THA during induction heating. The investigated work piece, as well as the used inductor, were modelled according to the experimentally used pieces (Figure 1a).

The simulation was designed considering two main aims. Firstly, minimisation of the computational time. For this reason, meshing was performed thoughtfully to ensure both reliable results and reasonable computational time. The mesh of the work piece consisted of 2700 volume tetrahedral elements, whereas the mesh of the inductor consisted of 21,314 nodes in total (Figure 1b).

The second aim was minimisation of the real heating time. For this reason, the simulation (and the following experiment) involved two-step heating. The first step, defined with the current of 68 A and frequency of 16.5 kHz, was designed to provide the quickest possible achievement of the swaging temperature of 900 °C, whereas the second step ensured homogenisation of the temperature throughout the cross-section, i.e., minimised temperature gradient from the surface to the axis of the work piece. Once the surface temperature of 900 °C had been reached, the second heating step, defined by a current of 50 A and a frequency of 15.8 kHz, began. However, possible development of residual stress resulting from the variable heating rate and occurring temperature gradients was also considered. The entire heating process was optimized for the maximum temperature of 900 °C not to be exceeded by more than 20 °C. To ensure thorough evaluation and control of the process, three locations at the axial longitudinal cut through the work piece, depicted as sensors 1, 2, and 3 (Figure 1c), were selected and monitored during the entire heating process.

**Figure 2:**
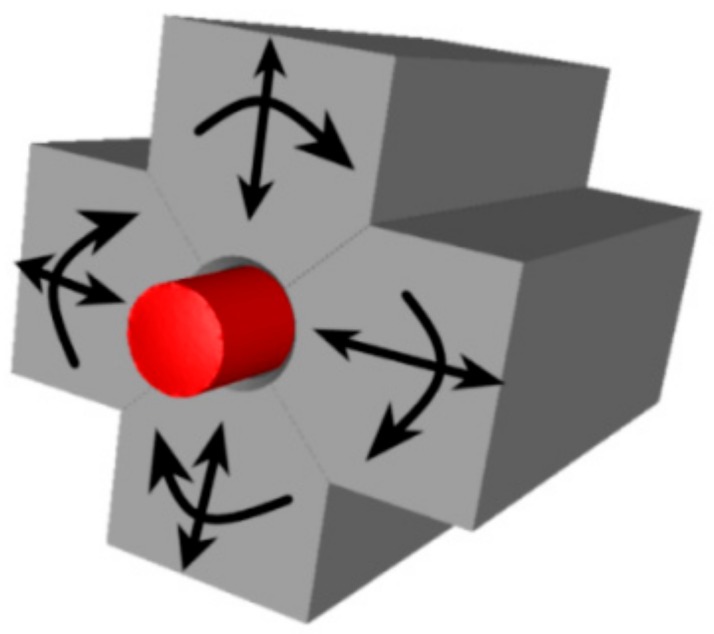
Schematic depiction of setup of rotary swaging.

The FORGE NxT software was also used to perform numerical prediction of deformation behaviour of the investigated 93W6Ni1Co alloy. The conditions for the simulation were selected to correspond to the real ones, and the entire simulation consisted of two simulation steps, corresponding to the real two swaging passes on which were modelled. The assembly for the simulation consisted of four swaging dies and the work piece, which was fed continuously towards the swaging head with the dies (Figure 2). The dies were created as shells, while the work piece was designed as a full deformable body with the mesh element size of 0.3 mm. Deformation behaviour of the material was characterized via the Haensel-Spittel equation, presented as Equation (1),

(1) $$\sigma =A\exp(m_{1}T)T^{m_{9}}\varepsilon^{m_{2}}\exp\left(\frac{m_{4}}{\varepsilon }\right){\left(1+\varepsilon \right)}^{m_{5}T}\exp\left(m_{7}\varepsilon \right){\dot{\varepsilon }}^{m_{3}}{\dot{\varepsilon }}^{m_{8}T},$$

where $\dot{\varepsilon }\;$ is the equivalent strain rate (s^−1^), ε is the equivalent strain (-), T is the temperature (°C), and A, m_1_, m_2_, m_3_, m_4_, m_5_, m_7_, m_8_, and m_9_ are regression coefficients, the values of which were set based on regression analyses of plastometric test results (A_1_ = 1447.002738, m_1_ = −0.009, m_2_ = 0.0895, m_3_ = 0.0044, m_4_ = −0.0069, m_5_, m_7_, m_8_, and m_9_ were equal to 0). The dimensions of the test specimens for the plastometric tests were 10 mm in diameter and 150 mm in length. Friction was defined via the Coulomb law, μ = 0,1, and the specific parameters for the simulation were the following: Poisson coefficient 0.3, specific heat 130 J·kg^−1^K^−1^, density 18.5 g·cm^−3^, and thermal conductivity (λ_20_ = 149.3 W·m^−1^K^−1^, λ_900_ = 85.7 W·m^−1^K^−1^).

### 2.2. Experimental Verification

The selected 93W6Ni1Co pseudo-alloy with the chemical composition of 92.6 wt.% W, 5 wt.% Ni and 2.40 wt.% Co (determined by SEM-EDX analysis) was prepared from powders with the mean grain size of 2.78 μm, containing ~<13 ppm of Fe, Mo, Cr, Al, Ca and other impurities. The process involved mixing of powders, cold isostatic pressing at 400 MPa, and subsequent sintering at 1525 °C for 20 min under a protective atmosphere followed by quenching in water. The protective atmosphere is hydrogen during sintering and argon during cooling. This preparation procedure was performed at ÚJP Praha a.s.

The sintered pieces were subsequently processed via rotary swaging, the initial temperatures for which were 20 °C (cold swaging) and 900 °C (warm swaging). The swaging temperatures were selected based on our previous experimental studies [3,31]. The room temperature was selected in order to maximize the possible achievable strength and to investigate, whether the material would maintain reasonable plasticity. The temperature of 900 °C was selected as the highest applicable temperature, since our previous studies showed that processing at temperatures higher than 900 °C resulted in massive oxidation causing rapid embrittlement during processing of the WNiCo. The temperature control during induction heating was provided by optical pyrometer (surface temperature), and a couple of thermocouples of type K (one on the surface, another in the work piece axial region). The initial diameter of the sintered pieces was 30 mm, the length was 100 mm. Both were gradually swaged down in two consequent reduction steps to the final diameter of 20 mm. Such swaged pieces are suitable, e.g., for the fabrication of kinetic penetrators [26]. The following structural analyses performed on cross-sectional cuts of the swaged pieces focused primarily on the influence of the applied deformation temperature on (sub)structure development and residual stress. The observations were performed via scanning electron microscopy (SEM EBSD analyses). The cut samples were mechanically ground on SiC papers and subsequently polished using Eposil F substance (Saphir 520 device, ATM, Germany). The EBSD analyses were performed in the sub-surface sample region, 1 mm from the outer rim of the swaged-rod, with the scan step of 0.25 µm using a Tescan Lyra 3 equipment (TESCAN Brno s.r.o, Brno, CZ) with a NordlysNano EBSD detector (Oxford Instruments, Abingdon-on-Thames, UK). The scans were evaluated using the ATEX (Win10 version) software [32]. Residual stress was characterized via analyses of internal grains misorientations in the scale from 0° to 15° (rainbow colour distribution). TEM images were acquired on ion polished thin foils with a JEOL 2100F (JEOL, Akishima, Tokio prefecture, Japan) device. The tensile tests were performed with the strain rate of 10^−3^ using a Testometric M500-50CT testing machine (Testometric Co. Ltd., UK) on 150 mm long testing samples with circular cross-sections.

## 3. Results

### 3.1. FE Analyses

#### 3.1.1. Induction Heating

**Figure 3:**
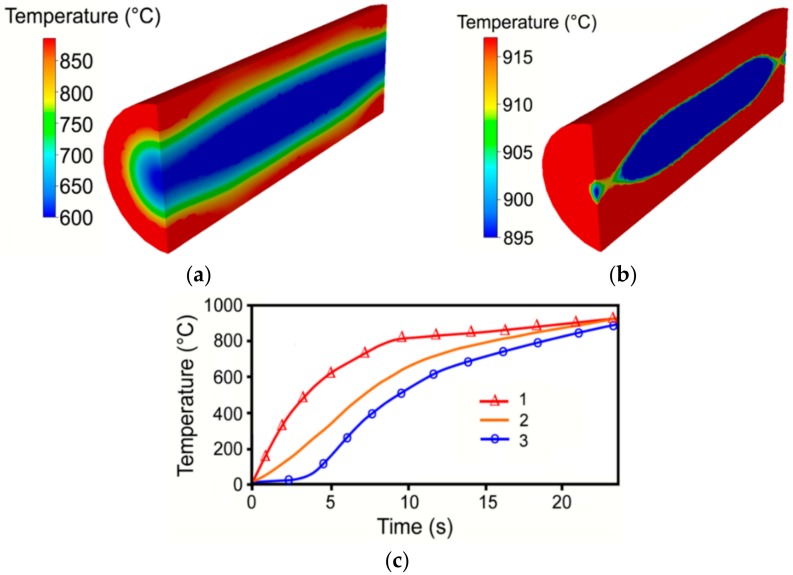
Temperature field during induction heating in time: 12 s (**a**); 24 s (**b**); distribution of temperature in monitored sensors (**c**).

As shown by the FEA results, induction heating imparted the development of zones featuring various temperatures, similar to conventional heating in furnaces. As documented by Figure 3a, depicting the temperature field throughout the work piece in the moment of achieving the surface temperature of 900 °C (heating time 12 s), the difference between the temperatures in the surface and axial work piece regions was up to 350 °C in this moment. Nevertheless, the temperature gradient decreased rapidly as heating continued. The results showed that the heating time necessary to heat the THA work piece to the required temperature of 900 °C with the maximum allowable deviation of 20 °C and, at the same time, to provide homogeneous temperature distribution throughout the entire work piece, was 24 s (Figure 3b,c).

The temperature distribution depicted in Figure 3b shows that the temperature field across the cross-section was not homogeneous along the work piece, especially during the second heating step. This is also confirmed by Figure 3c, which shows the differences between the temperatures in the individually monitored sensors. This phenomenon was a result of the geometry of the used inductor. The temperature increase was the highest in the surface work piece region up to the heating time of 10 s; the heating rate was lower in the other two monitored locations during that time. The lowest heating rate was monitored in the axial work piece area. The second heating step then imparted rather unexpected material behaviour, the heating rate in the peripheral work piece area decreased, while the other two monitored areas kept more or less the same heating rate. This behaviour finally introduced a decrease in the temperature gradient, i.e., homogenisation of the temperature field throughout the work piece cross-section, which is documented by Figure 3c.

#### 3.1.2. Deformation Behaviour

**Figure 4:**
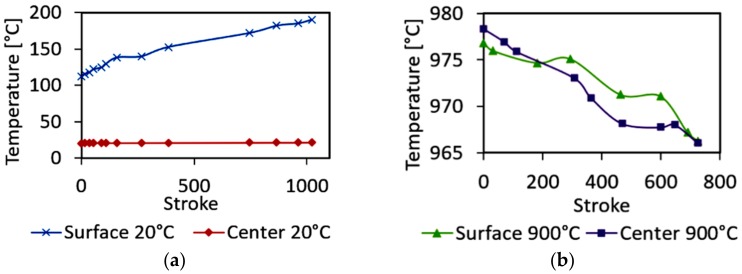
Temperature developments in surface and axial regions of the cold-swaged piece (**a**) and the warm-swaged piece (**b**).

The predicted developments of temperature in the surface and axial regions during processing of both the work pieces, swaged at 20 °C and 900 °C, are depicted in Figure 4a,b, respectively. As shown in the Figures, the initial temperatures for both the swaged pieces were higher than the original swaging temperatures due to the previous deformation history (first swaging pass).

As can be seen from Figure 4a, the temperature in the surface area increased gradually to almost 200 °C during cold swaging, whereas the temperature in the axial area remained more or less constant. On the other hand, the warm-swaged piece exhibited a decrease in temperature during swaging; the temperature decrease rate was comparable for both the surface and the axial areas (Figure 4b). However, multiple affecting phenomena need to be considered for the surface areas of the swaged pieces. The surface temperature increases due to the effect of friction with the rotating dies being in contact with the surface. On the other hand, the dies are cooler than the swaged piece, which causes a certain portion of the generated heat to dissipate via the swaging dies.

**Figure 5:**
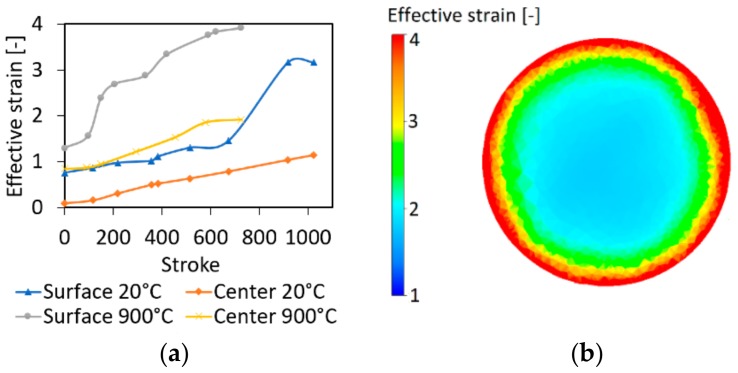
Developments of imposed effective strain in surface and axial regions of both swaged pieces (**a**); effective strain across cross-section of warm-swaged piece (**b**).

Figure 5a depicts the predicted developments of the imposed effective strain in the surface and axial regions of both the swaged pieces. The comparison of both the curves for the cold-swaged piece shows that the imposed strain was substantially higher in the surface region of the swaged piece, where it reached a maximum value of almost 4. The figure also shows that the maximum effective strain in the axial region of the cold-swaged piece reached to the value of 1.2. On the other hand, warm swaging imparted an increase in the imposed effective strain, which reached and even slightly exceeded the value of 2, approximately in the middle of the swaging pass. Mutual comparison of the effective strain developments for both the swaged pieces reveals that the strain gradient from the surface towards the swaged piece axis was substantially lower for the warm-swaged piece. Nevertheless, the maximum imposed effective strain was comparable for both.

The distribution of the effective strain across the cross-sectional cut through the warm-swaged piece is depicted in Figure 5b. The figure shows that the imposed effective strain reached a value of 4 at the very periphery of the swaged piece. The imposed strain then gradually decreased towards the axial region where it reached the values of approximately 2.

**Figure 6:**
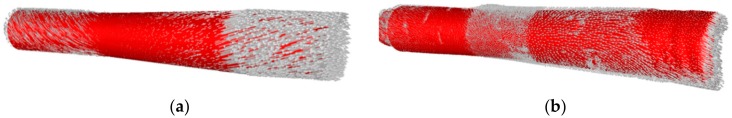
Vectors of material flow for the cold-swaged piece (**a**) and the warm-swaged piece (**b**).

Figure 6a,b depicts the material flow during swaging of the cold and warm swaged piece, respectively. Given by the incremental nature of the swaging process and rotary movement of the swaging dies, material flow during swaging is variable and quite complex. The material flow was different for both the swaged pieces, and also changed its character during the individual swaging pass. At the beginning of swaging, the axial material flow dominated for both the cold and warm swaged pieces; however, the plastic flow vectors were flexed for both the pieces. With continued swaging, the material flow changed its character, i.e., it rotated backwards in the neutral zone, due to the effect of the already swaged deformation strengthened material volume. For the cold-swaged piece, the neutral zone was located in the reduction zone (Figure 6a), while for the warm-swaged piece, the position of the neutral zone shifted towards the feeding zone of the swaging dies (Figure 6b).

**Figure 7:**
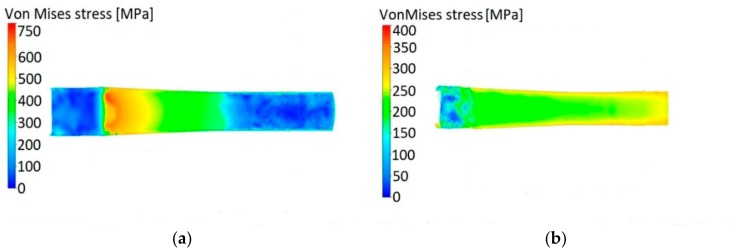
Stress intensity in axial longitudinal cut depicted via Von Mises stress for the cold-swaged piece (**a**) and the warm-swaged piece (**b**).

The finally evaluated predicted parameters were stress-related ones, stress intensity during swaging, and residual stress. Figure 7a,b depicts stress intensity via Von Mises stress throughout the axial longitudinal cut for the cold and warm swaged piece, respectively. As can be seen in Figure 7a, the stress intensity during cold-swaging increased to 750 MPa, while for the warm-swaged piece, the maximum stress intensity reached approximately 350 MPa.

**Figure 8:**
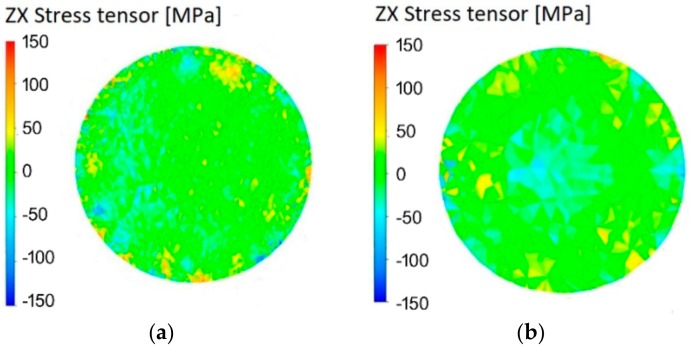
Predicted residual stress intensity across cross-section of the cold-swaged piece (**a**) and the warm-swaged piece (**b**).

Stress can be present in the material not only during the actual processing, but residual stress can also be present after the material left the swaging dies’ reduction zone. Figure 8a,b depicts the predicted distribution of residual stress across the cross-section of the cold and warm swaged piece, respectively. Mutual comparison of the figures reveals similar distribution of residual stress within both of the swaged pieces, its intensity corresponding to the intensity of the imposed effective strain for both pieces. Deeper insight into the residual stress distribution was subsequently provided via experimental analysis.

### 3.2. Structure Analyses

**Figure 9:**
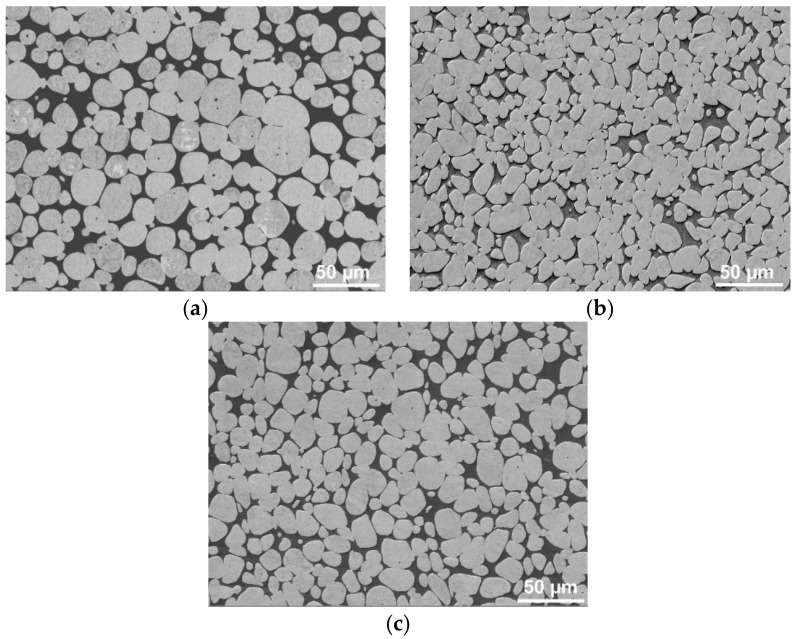
SEM-BSE structure scans for sintered state (**a**); cold-swaged piece (**b**); warm-swaged piece (**c**).

Figure 9a–c shows the SEM-BSE scans of the tungsten pseudo-alloy after sintering, cold-swaging, and warm-swaging, respectively. As can be seen, the sintered state exhibited individual round-shaped W agglomerates surrounded by the NiCo matrix (Figure 9a). The structures after cold and warm swaging (Figure 9b,c) exhibited visible differences in the shapes of the agglomerates. While the grains of both the matrix and tungsten deformed significantly during cold swaging due to the substantial imposed shear strain, the elevated temperature during warm swaging decreased the matrix flow stress, which caused the softer matrix to preferentially consume the imposed strain. For this reason, the tungsten agglomerates remained more or less of round shapes.

**Figure 10:**
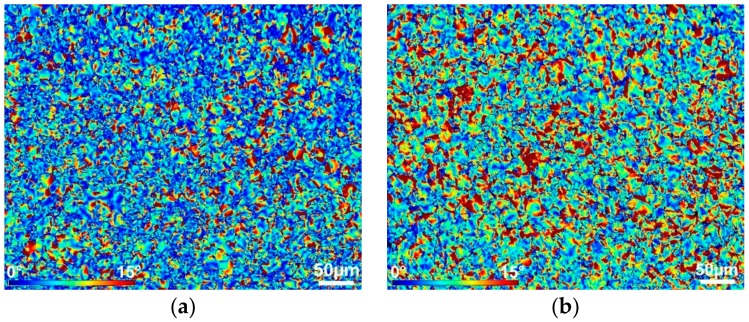
Integral grain misorientations for ChemEngineering-610706 cold-swaged piece (**a**) and the warm-swaged piece (**b**).

Figure 10a,b shows residual stress depicted via internal misorientations from 0° to 15° for the cold and warm swaged pieces, respectively. For both pieces, the presence of residual stress was detected in the peripheral regions of the tungsten agglomerates. This phenomenon can be attributed primarily to substructure development and accumulations of dislocations, as proven by the further discussed TEM structure characterisation.

As shown by the computational analyses of material flow, the increased temperature of 900 °C imparted a more intense plastic flow, which imparts a higher probability of fixation of elastic strain within the warm-swaged material. In other words, the warm-swaged piece (Figure 10b) exhibited a higher volume of locations featuring high internal misorientations, i.e., internal stress, than the cold-swaged piece (Figure 10a). As can be seen from Figure 9b, the misorientations occurred not only at the peripheries of the agglomerates, but also within the more ductile NiCo matrix.

**Figure 11:**
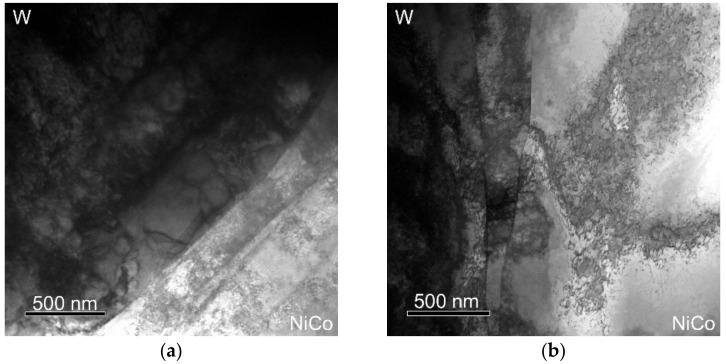

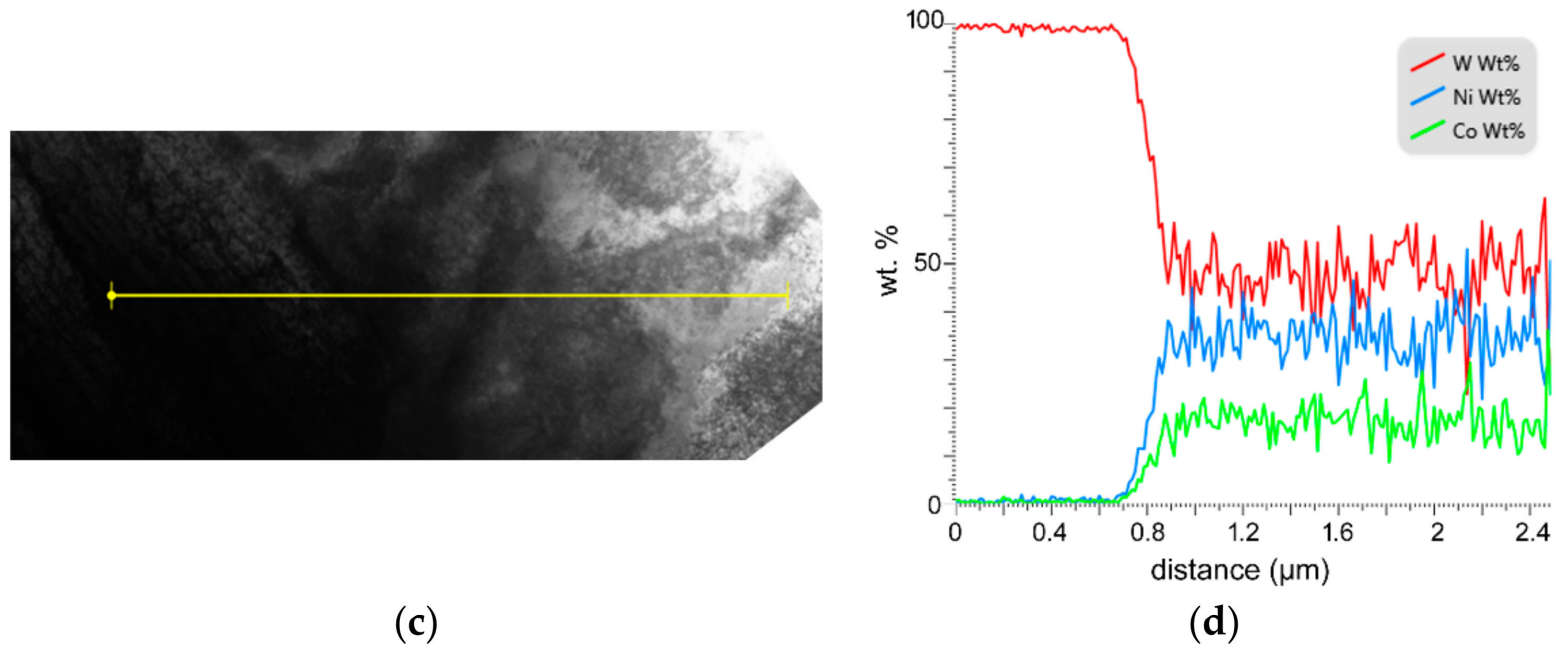
TEM scan showing agglomerate/matrix interface for the cold-swaged piece (**a**) and the warm-swaged piece (**b**); measured line scan area within the warm-swaged piece (**c**); and chemical composition of investigated line scan (**d**).

Figure 11a,b depicts TEM scans of the cold- and warm-swaged pieces, respectively. As can be seen, the cold-swaged piece exhibited distinctive interfaces between the tungsten agglomerates (dark area) and the NiCo matrix (light area). On the other hand, the warm-swaged piece exhibited diffusion of tungsten to the NiCo matrix; the areas within the matrix with increased tungsten content can be seen in the figure as “dark shadows” within the light matrix area. To confirm the presence of tungsten within the matrix, a line scan measuring chemical composition through such a darker shadow within the matrix was carried out. Figure 11c depicts the area in which the line scan was carried out (depicted by the straight line). Figure 11d then shows the results of the line scan as regards the chemical composition of the investigated area. As is evident, the size of the interface between the agglomerate and the matrix after warm swaging and the content of tungsten within the matrix in the vicinity of the agglomerates reached up to 50 wt.%.

### 3.3. Mechanical Properties

The mechanical properties of both the swaged pieces were evaluated via tensile tests; the ultimate tensile strength (UTS) and elongation till failure for all the investigated pieces are summarized in Table 1.

As can be seen, the powder-based sintered material exhibited the lowest recorded UTS of approximately 860 MPa; however, the elongation till failure reached up to ~20%. The highest strength was recorded for the cold-swaged piece, which it exhibited a UTS of 1828 MPa after the second swaging pass. The UTS after warm swaging increased too, but the UTS value was slightly lower than after cold swaging (1650 MPa vs. 1828 MPa). The fact that the increase in UTS occurred at the expense of plasticity for both the swaged pieces needs to be stressed. In other words, the decrease in plastic properties after swaging was evident in both cases (especially with higher imposed strain). However, the more favourable mutual combination of strength and plastic properties was achieved after warm swaging.

## 4. Discussion

The numerical simulations revealed certain differences in the material behaviours of both the work pieces during swaging. For successful swaging of the cold-swaged piece, no preheating was necessary, which makes the swaging process more effective from the viewpoint of processing time. On the other hand, induction heating of the warm-swaged piece is considerably quicker when compared to heating in a furnace and homogeneous heating of the work piece to the swaging temperature only took 24 s. Nevertheless, certain temperature gradient between the surface and axial regions of the work piece developed during heating. The gradient was primarily dependent on the heating time; it was most significant at the beginning of heating and diminished by the time heating was finished.

Among the advantages of induction heating is also that it can favourably be placed directly in front of the swaging machine, which, however, directly affects the material characteristics [33,34], especially the complex material flow. Continuous heating keeps the unswagged work piece in front of the swaging head at the swaging temperature of 900 °C, which introduces gradients in mechanical properties, especially flow stress, between the swaged and unswaged material volumes. As shown by the predicted plastic flow vectors, gradients in temperature-related material properties influenced significantly localization of the neutral zone, the plastic flow vectors in which changed their orientations.

Regardless the effect of induction heating, the predictions revealed temperature variations during processing for both the swaged pieces. The cold-swaged piece exhibited gradually increasing difference in temperatures in the surface and axial swaged piece regions (up to almost 200 °C). Such behaviour is typical for cold swaging technology; the surface temperature of the swaged piece increases primarily due to the effect of direct contact of the surface with the swaging dies, i.e., friction effect [35]. Nevertheless, this effect is reduced by the heat transfer through the cold swaging dies. Warm swaging also introduced a certain increase in temperature, primarily in the work piece surface region, where the maximum temperature increased to 990 °C. In addition to the effect of friction, the temperature increase during swaging can also be attributed to deformation heat generated by the influence of the imposed shear strain [36].

The material characteristics during swaging are primarily influenced by the effect of the swaging force, featuring two main components: tangential and axial [29]. Whereas the axial swaging force component contributes to the gradual elongation of the swaged piece and provides the swaged product with its final shape, the tangential swaging force component imparts intensive shear strain, resulting in structure changes, introducing an increase in the actual processing temperature, imparting flexure of the plastic flow vectors, but also imparting the gradient of the imposed effective strain from the swaged piece surface region towards its axial region; the effective strain is the highest in the surface swaged piece region, the effect of the rotating dies introducing shear strain, which is the most intense. This phenomenon was evident especially for the cold-swaged piece, the flow stress for which was aggravated. During warm swaging, the flow stress of the THA decreased, reducing the effective strain gradient across the swaged piece cross-section, but also resulted in the higher total imposed effective strain.

The flow stress, together with the differences in the plastic flows of both the swaged pieces, also non-negligibly influenced the stress intensity, and consequently the load on the swaging machine. The predicted stress intensity, pointing to the resistance of the swaged material against deformation, was significantly higher for the cold-swaged piece. The predicted distribution of residual stress was more or less comparable for the cold and warm swaged piece. Nevertheless, the experimental observations showed micro-differences in the distribution of residual stress within the NiCo matrix. The results showed that both the swaged pieces exhibited the presence of residual stress in the peripheral areas of tungsten agglomerates. As proven by the transmission electron microscopy analyses, this phenomenon could primarily be attributed to substructure development within the agglomerates. However, the more intense plastic flow supported by the elevated swaging temperature introduced the presence of residual stress, i.e., unrelaxed elastic deformation, within the matrix of the warm-swaged piece. The primary reason for such behaviour lay in the intense plastic flow imparted by the increased swaging temperature. The harder tungsten agglomerates featuring lower formability acted as “transferring agents” of the imposed strain during swaging. During processing, mutual distances between the individual agglomerates decrease, i.e., the volume of the ductile matrix between the hard agglomerates gradually decreases, by the effect of which the interactions of the individual agglomerates become more intense. Moreover, the occurring diffusion of tungsten to the NiCo matrix, which intensifies with increasing processing temperature, should also be considered. By the effect of this phenomenon, the matrix hardens, and residual stress develops, especially at the W/NiCo interfaces. In other words, the matrix then involves locations featuring varying chemical compositions, imparting local differences within the matrix, and consequently introducing strain gradients resulting in the presence of residual stress.

## 5. Conclusions

The study focused on the numerical prediction of the deformation behaviour of a 93W6Ni1Co tungsten heavy alloy rotary swaged at 20 °C and 900 °C. The study was supplemented with numerical investigations of the applied induction heating, and experimental observations of residual stress and substructure development within the swaged pieces.

The predictions showed the overall time for heating of the THA to 900 °C to be 24 s. The induction heating promoted shifting of the neutral zone the plastic flow vectors in which reverse primarily by the effect of flow stress gradient between the swaged and unswagged materials.

Warm swaging at 900 °C imparted a more homogeneous distribution of the imposed strain. However, for both the swaged pieces, the highest strain was observed in their surface regions, which were directly affected by the swaging dies. This finding corresponded to the predicted distribution of temperature throughout the swaged pieces; for both, the temperature increased in their surface regions primarily due to the development of deformation heat.

Both the swaged pieces exhibited the presence of residual stress in the peripheral areas of tungsten agglomerates, which could primarily be attributed to substructure development. However, the supported plastic flow of the warm-swaged piece also introduced the presence of residual stress, i.e., unrelaxed elastic deformation, within the warm-swaged piece matrix, and diffusion of W to the NiCo matrix. The UTS was the highest for the cold-swaged piece (1828 MPa), and slightly lower for the warm-swaged piece (1650 MPa); this phenomenon can primarily by attributed to the occurrence of softening of the matrix.

## Figures and Tables

**Table 1:** Mechanical properties resulting from tensile tests.

Swaging Pass	Swaging Temperature (°C)	UTS (MPa)	Elongation (%)
0	-	857 ± 12	18.3 ± 3.5
1	20	1412 ± 21	9.2 ± 2.4
2	20	1828 ± 17	5.6 ± 1.3
1	900	1010 ± 19	23.4 ± 3.2
2	900	1650 ± 15	7.8 ± 1.5
